# A Synergistic Approach to Sustainable Packaging: Exploring Mater-Bi^®^/PHBV Multilayer Systems

**DOI:** 10.3390/polym18060692

**Published:** 2026-03-12

**Authors:** Similoluwa Oluwaferanmi Orisaya-Taiwo, Loredana Incarnato, Eugenio Amendola, Stefania Dello Iacono, Luciano Di Maio

**Affiliations:** 1 Department of Industrial Engineering, University of Salerno, Via Giovanni Paolo II, 84084 Fisciano, SA, Italy; sorisayataiwo@unisa.it (S.O.O.-T.); lincarnato@unisa.it (L.I.); 2 Institute for Polymers, Composites and Biomaterials, National Research Council, P.le Enrico Fermi 1, 80055 Portici, NA, Italy; stefania.delloiacono@cnr.it

**Keywords:** blend, multilayer, co-extrusion, film blowing, mechanical properties, barrier properties, sealability, food packaging

## Abstract

In this study, multilayer films were produced using a co-extrusion process involving two complementary bioplastics: Poly(3-hydroxybutyrate-co-3-hydroxyvalerate) (PHBV) and Mater-Bi^®^. PHBV is recognized, among biodegradable polymers, for its excellent oxygen barrier properties, but is also known for being brittle, non-sealable, and difficult to process. Conversely, Mater-Bi^®^ has good processability, low stiffness, and high ductility, but exhibits poor barrier properties. The research investigated the barrier, mechanical, sealing, and optical properties of the films to evaluate how variations in layer structure and processing parameters influenced their overall performance. The results showed that the addition of the PHBV layer, both as a neat polymer and as a blend with Mater-Bi^®^, significantly reduced oxygen and water vapor permeability. Additionally, Mater-Bi^®^ was found to be essential for providing the elasticity and sealability required for the multilayer films. The study also demonstrated that layer thickness plays a critical role in property tailoring.

## 1. Introduction

Trends in the modern food packaging industry are centered on two distinct goals: enhancing food safety and environmental responsibility, particularly through the reduction in plastic packaging waste. This has led to growing interest in biopolymers, which are seen as a viable alternative to synthetic, petrochemical-derived polymers. The fundamental characteristics of biopolymers—their inherent biodegradability and biocompatibility—enable them to prevent environmental contamination while maintaining the preservation and quality assurance needed for food products [1,2]. The use of biodegradable flexible packaging holds significant potential for environmental protection, as it directly addresses critical global challenges such as packaging waste generation and the persistent accumulation of non-biodegradable plastics in terrestrial and marine ecosystems. These materials offer a substantial advantage over conventional plastics, particularly given the well-documented issue of toxic and carcinogenic monomers and oligomers leaching from plastic surfaces and structures into the environment [3]. The adoption of bio-based and biodegradable or compostable materials for food packaging applications is therefore a key strategy for mitigating the adverse environmental impacts associated with traditional plastics, a perspective that has promoted a surge of scientific and industrial interest in bioplastics [4]. The imperative for their adoption reflects that packaging constitutes roughly 40% of all plastic products, making the transition to sustainable alternatives crucial for the mitigation of pervasive environmental risks.

However, despite their clear advantages, the practical application of these polymers in packaging remains constrained by significant technological limitations. The most significant is their inadequate gas and moisture barrier performance, a critical drawback that severely restricts their viability for use in demanding applications such as food packaging, where product preservation and shelf life are paramount. In response to this challenge, decades of academic and industrial research have focused on developing strategies to enhance the performance of these materials. Key approaches include melt blending and multilayer co-extrusion, which involves combining different polymers to create a composite material that utilizes the superior properties of each component. These strategies represent a pathway toward overcoming the functional limitations of current biodegradable polymers, enabling their broader integration into a circular economy for packaging materials [5,6].

Poly(3-hydroxybutyrate-co-3-hydroxyvalerate) (PHBV) is a copolymer from the polyhydroxyalkanoates (PHAs) microbial polyester family. PHBV is an aliphatic co-polyester synthesized through bacterial fermentation of sugars and lipids. It is receiving increasing attention due to its complete biodegradability across a range of environmental conditions, its high mechanical stiffness (high tensile elastic modulus) and low oxygen and moisture permeability [7,8,9,10,11]. PHBV exhibits a limited thermal processability, being suitable for techniques such as injection molding and hot compression molding and a very low processability for film extrusion. Moreover, large-scale use of PHBV in packaging is restricted by its brittleness, thermal instability, and narrow processing window.

Mater-Bi^®^ is a family of biodegradable and compostable thermoplastic starches (TPSs) developed by Novamont. It is widely used in different grades as a commercial compostable polymer due to its very good processability to produce blown films. Its main components are maize starch and a variety of synthetic chemicals, including hydrophilic materials and natural plasticizers. While the precise formulation remains proprietary, existing scientific literature emphasizes the potential of TPS-based blends for film production [12]. In many cases, the immiscibility issues of the different components of the blends have been studied and addressed by the use of compatibilizers to prevent phase separation and to ensure strong adhesion between phases [13,14]. Starch-based films have emerged as a prominent focus of research due to their promising application potential, but when compared to traditional petroleum-derived films, they exhibit significant limitations, such as high susceptibility to water vapor and comparatively lower mechanical strength.

Therefore, Mater-Bi^®^ and PHBV exhibit complementary properties that make them promising candidates for combined use in biodegradable material applications: Mater-Bi^®^ offers excellent processability and ductility, making it suitable for various manufacturing techniques. In contrast, PHBV provides superior barrier properties but suffers from limited processability and inherent brittleness. The integration of these two materials could potentially balance their respective strengths and overcome individual limitations.

Polymer blending is a widely adopted and cost-effective method used in industry to improve the properties of biopolymers. There is extensive literature on biodegradable polymeric blends with particular focus on improving the mechanical properties and toughness of particularly brittle polymers such as polyhydroxyalkanoates (PHAs). V. Beber et al. [15] have studied the blends of polyhydroxybutyrate (PHB) with poly(butylene adipate-co-terephthalate) (PBAT) and a natural filler, demonstrating an increased ductility and processability as the proportion of PBAT rises. M. Jordá-Reolid [16] reports that the addition of polybutylene succinate (PBS) as a blend helps reduce the rigidity of biodegradable polymers such as polylactic acid (PLA) and PHAs. Ternary blends of PLA/poly(3-hydroxybutyrate-co-3-hydroxyvalerate) (PHBV)/PBS at 60/30/10 display optimal performance with a good balance of stiffness, toughness, and thermal resistance [17]. Another example of property tailoring is represented by the PHB/PLA blend, which demonstrates a significant reduction in oxygen transmission rate, a decrease in wettability, and an improvement in mechanical properties [18]. Another study shows that films produced via extrusion of PHBV/PBAT (40:60) blends with varying amounts of organically modified nano clay exhibit improved elongation at break, tensile strength, and modulus—along with enhanced barrier properties—making them suitable for flexible packaging [19].

In contrast to melt blending, multilayer co-extrusion stands as a distinct and highly scalable manufacturing process that has proven effective in producing high-performance, petroleum-based packaging films. This technique is essential for developing films with superior barrier properties against gases and moisture. The fundamental principle of co-extrusion technology is the synergistic integration of the properties of different polymers within a single, cohesive, multilayered structure. For instance, a multilayer film can be engineered to combine the excellent oxygen barrier of ethylene vinyl alcohol (EVOH), the moisture barrier of polypropylene, and the heat-sealing capability of polyethylene, thereby achieving a tailored balance of properties optimized for specific packaging applications. With respect to melt blending, the co-extrusion process does not present the limitations of polymer compatibility and miscibility and allows for overcoming the limited processability of some polymers. The industrial relevance of co-extrusion is related to the customization of properties without full material blending and cost efficiency by placing more expensive or performance-critical materials only where needed, with very low thicknesses.

However, the multilayer co-extrusion process requires specialized equipment, such as multi-extruder systems and feed blocks or multiple manifold dies, to ensure the uniform and consistent lamination of the different polymer layers, including eventually the tie layers needed for delamination prevention [20]. Many papers address the issue of property enhancement by combining different biodegradable polymers via co-extrusion. For instance, the co-extrusion of PLA/poly(butylene succinate-co-butylene adipate) (PBSA) systems in a thickness ratio of 80:20 results in a more barrier-enhanced and ductile film than a film made solely of PLA [21]. Biodegradable bilayer films of Ecovio/PLA 4060 and PBS/PLA4032 (70:30) were investigated, where the inner and outer layers, respectively, were co-extruded to give a high barrier film for long-term frozen food storage [22]. In another work, PLA/Mater-Bi^®^ bilayer films were produced using co-extrusion and compared to the respective monolayer films, which showed good optical and mechanical properties [23], whereas bilayer films of PLA4032/PHB blend (70:30) (outer layer) and Ecovio/PLA4060 (80:20) (inner layer) were studied to give a reduction in oxygen permeability and ductility [24].

Thus, although melt blending is a standard approach for toughening brittle biopolymers [15,16,17,18], it often entails a significant trade-off in barrier performance due to the dilution effect and the irregular morphology inherent in random phase dispersion. In contrast, multilayer co-extrusion overcomes these limitations by maintaining the different polymers as discrete, continuous layers, thereby fully exploiting their intrinsic properties within a synergistic composite architecture.

While Mater-Bi^®^ and PHBV have been extensively characterized individually or in blends with other biodegradable polymers like PLA and PBAT, their combination via multilayer co-extrusion remains largely unexplored. Currently, there is a scarcity of literature regarding the thermomechanical compatibility of starch-based and PHA-based systems. However, in this study, co-extruded films exhibited no signs of delamination. This physical integrity aligns with findings by Avella et al. [25], who noted that PHAs often demonstrate inherent compatibility with other biodegradable polyesters, potentially facilitating robust interlayer adhesion without the requirement of a tie layer.

The present research is thus aimed at exploring the following technological innovations:

(a) Processing synergy: The high melt strength of Mater-Bi^®^ allows it to act as a rheological ‘carrier’ and stabilizer for the bubble during the film-blowing process. This strategy effectively overcomes the processing instability and narrow thermal window of PHBV—limitations that typically hinder its transformation into flexible films when processed alone or in simple blends.
(b) Properties synergy: Mater-Bi^®^ exhibits high flexibility, good sealability and high ductility. However, it suffers from low mechanical strength and quite low barrier properties. PHBV, on the other hand, demonstrates high crystallinity, good gas barrier properties, and relatively high tensile strength, but is more brittle and thermally sensitive during processing. Thus, a multilayer structure via co-extrusion allows for a synergistic combination of these properties, where the PHBV layer acts as a gas or aroma barrier, whereas the Mater-Bi^®^ layer provides ductility, sealing ability, and improved processability. Given that both polymers are independently industrially scalable, exploring their co-extrusion feasibility has immediate commercial and environmental relevance.
(c) Structural and environmental efficiency: Unlike conventional petroleum-based multilayers that rely on synthetic tie layers [20], this work is aimed at developing a simplified, fully biodegradable, and industrially scalable architecture that balances the high flexibility and sealing ability of Mater-Bi^®^ with the superior crystallinity and barrier performance of PHBV. The successful production of a stable structure via multilayer blown film co-extrusion serves as a functional validation of interfacial compatibility; the total absence of delamination indicates that the system achieves sufficient adhesion without the requirement of additional compatibilizers or tie layers.

Thus, this study fills a critical gap in the field of sustainable packaging by assessing the co-extrusion feasibility of Mater-Bi^®^/PHBV/Mater-Bi^®^ structures through a comprehensive evaluation of their properties in order to provide a circular-economy-compliant alternative to traditional plastic films.

## 2. Materials and Methods

### 2.1. Materials

The materials used for this study are a film-blowing grade of Mater-Bi^®^ (EF05B), a product of Novamont (Novara, Italy) with a density of 1.28 g/cm^3^ and a melting temperature of 125 °C, and ENMAT Y1000P PHBV, with a density of 1.25 g/cm^3^ and a melting temperature of 172 °C, acquired from Ningbo Tianan Materials Co., Ltd. (Ningbo, China). All materials were dried before use. The blend was prepared using Mater-Bi^®^/PHBV 80/20 *w*/*w*.

### 2.2. Film Blowing

**Figure 1:**
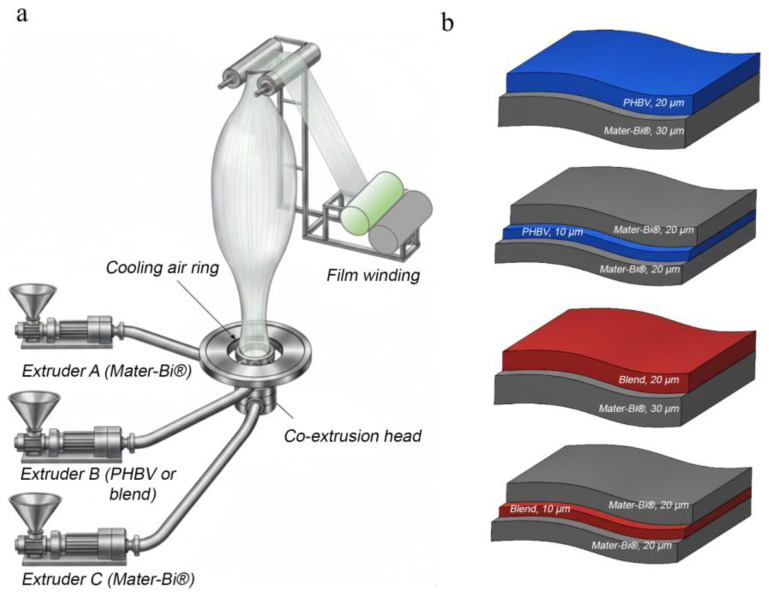
Film production through film-blowing process (**a**); bi- and trilayer films with thickness of 50 µm (**b**).

The PHBV and Mater-Bi^®^ pellets were dried under vacuum for 16 h at a temperature of 80 °C before processing. The film-blowing process was performed using a laboratory three-layer co-extrusion blown film apparatus, which included three single-screw extruders (Gimac, Castronno, Italy, Dscrew = 12 mm, L = 24D) connected to a three-layer co-extrusion head alongside a cooling and take-up system (Collin Film Blowing Line Type BL 50) (Figure 1a). The annular die of the extrusion head had D_in = 30 mm and D_ex = 32 mm. In the three-layer films of structure ABC, the internal and external layers A and C were composed of Mater-Bi^®^, while layer B was composed of neat PHBV or a blend of PHBV/Mater-Bi^®^ 20/80. The extrusion speed was set in the range of 30–45 rpm on the three extruders, and the take-up speed of the nip rolls ranged between 1 and 3 m/min. The blow-up ratio of the tubular film was maintained at 2. The temperature profile for the three extruders was set over four zones at values of 155, 165, 175, and 190 °C from the feeding hopper to the die. Monolayer films of PHBV and Mater-Bi^®^, multilayer films, and blends were studied. The PHBV, Mater-Bi^®^, and blend films were extruded at 45 rpm and collected at 2 m/min for a thickness of 50 μm. The multilayer samples were processed, setting the screw speed of each extruder at 30 rpm and a take-up speed of 3 m/min. The thickness of all the samples was 50 µm (Figure 1b).

### 2.3. Rheological Characterization

Rheological characterization was conducted using an Advanced Rheometric Expansion System (ARES) rotational rheometer (RHEO Service GmbH & Co. KG, Reichelsheim, Germany) equipped with a parallel plate geometry (25 mm diameter, 1.00 mm gap). All measurements were performed under a dry nitrogen atmosphere to suppress thermal and oxidative degradation during testing. To establish the linear viscoelastic region (LVR), strain sweep tests were first performed at an angular frequency of 1 rad/s and a temperature of 180 °C, with strain amplitudes ranging from 0.1% to 10%. The storage modulus (G′), loss modulus (G″), and complex viscosity (η*) remained nearly constant throughout this range, indicating a linear viscoelastic behavior response. Based on these results, a strain amplitude of 1% was selected for subsequent dynamic measurements. Dynamic frequency sweep tests were then performed at 180 °C under nitrogen, with the angular frequency varied from 0.1 to 100 rad/s. Rheological measurements were performed in duplicate for confirmation. Data analysis was carried out on a single representative curve.

### 2.4. Differential Scanning Calorimetry (DSC)

Thermal analysis was carried out using a differential scanning calorimeter Discovery DSC 2500 (TA Instruments, New Castle, DE, USA) under a constant nitrogen purge of 50 mL/min. A sample mass between 5 and 10 mg was selected to ensure optimal temperature control and signal accuracy. The heating/cooling rate was 10 °C/min. The thermal analysis protocol differed between pelletized and film samples. For Mater-Bi^®^ EF05B and PHBV ENMAT Y1000P pellets, three consecutive scans were performed: a first heating to eliminate unknown thermal history, followed by cooling and a second heating. Data reported for these materials came from the second heating and first cooling runs. In contrast, the 80/20 Mater-Bi^®^/PHBV blend and all biaxially oriented multilayer films (50 µm total thickness) were analyzed with a single heating–cooling cycle between −50 and 200 °C. The first heating scan of these film-blown samples directly shows thermal transitions resulting from the processing conditions, while the cooling scan characterizes crystallization from the melt. DSC measurements were performed in duplicate for confirmation purposes. Data analysis was carried out on a single representative curve. Transition temperatures and enthalpy values are reported as rounded to the nearest 0.5 °C and 1 J/g, respectively, as representative of the overall uncertainty of the measurements.

### 2.5. Permeability Tests

Water vapor permeability was evaluated using a Systech Illinois 7002 water vapor permeation analyzer (Systech Instruments, Princeton, NJ, USA) in accordance with ASTM F1249-90 [26]. Measurements were carried out at 23 °C and 50% relative humidity. The water vapor transmission rate (WVTR) was determined and used to calculate the permeability coefficient by accounting for the film thickness, as described by Equation (1):

(1) $$\mathrm{WVP}=\frac{\mathrm{WVTR}}{P*\left(R1-R2\right)}\cdot \;L$$

where *L* is the average film thickness (µm), *P* (bar) is the water vapor pressure at 23 °C, and *R*1 and *R*2 are the relative humidities (%) of the high- and low-humidity chambers, respectively.

Oxygen permeability was measured using a GTT gas permeabilizer (Brugger, München, Germany) at 23 °C and 25% relative humidity, according to ASTM D1434 standard [27] method. The tests were conducted with an oxygen flow rate of 80 mL/min on film specimens with an exposed area of 16 cm^2^. All measurements were performed in triplicate to ensure result reproducibility.

### 2.6. Mechanical Characterization

Mechanical tensile tests were performed on film specimens according to ASTM D882 [28]. A CMT 4000 Series tensile tester (SANS, Shanghai, China), equipped with a 1 kN load cell, was used for all measurements. Rectangular specimens (12.7 mm × 80 mm), cut along the machine direction, were tested. Two distinct crosshead speeds were used: the elastic modulus (E) was determined at a crosshead speed of 3 mm/min. Strength (σ_b_) and percentage elongation (ε_b_) at break were assessed at a speed of 300 mm/min. A set of 10 specimens was analyzed for each individual composition.

### 2.7. Heat-Seal Tests

The heat-sealing performance of the samples was evaluated following ASTM F2029-00 and ASTM F88-00 standards [29,30]. Rectangular specimens (2.5 cm × 19 cm) were heat-sealed using a Brugger model HSG-C heat sealer (Brugger, Munich, Germany) under a constant sealing pressure of 240 N for 1 s. The sealing temperature was systematically varied in 5 °C increments to identify optimal sealing conditions. After sealing, the samples were conditioned at 23 °C and 50% relative humidity for 48 h prior to mechanical testing. Seal strength was determined via tensile testing using a CMT 4000 dynamometer (SANS, China) at a crosshead speed of 300 mm/min until failure of the seal. The maximum load reported was considered the maximum seal strength of the film (expressed as N/25 mm). For each sample type, at least 10 measurements were performed to assess the reproducibility of the results.

### 2.8. UV-Vis Spectrophotometry

UV-Vis spectroscopic analysis of the film samples was performed using a Lambda 800 UV-Vis spectrophotometer (Perkin Elmer, Waltham, MA, USA), following the ASTM D1746 standard [31]. Film transparency was quantified by measuring the transmittance at 560 nm. Each sample was tested in triplicate to ensure reproducibility. The percentage transparency (*TR*%) was calculated according to Equation (2):

(2) $$\mathrm{TR}\left(\%\right)=\frac{\mathrm{TR}}{T0}\cdot \;100$$

where *TR* represents the transmittance of the specimen, and *T*0 is the baseline transmittance measured without the specimen in the light path.

## 3. Results

### 3.1. Rheology

**Figure 2:**
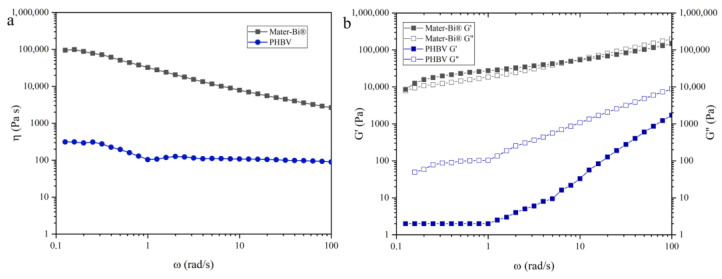
Complex viscosity (η*) (**a**), storage modulus (G′) and loss modulus (G″) (**b**) against the angular frequency (ω).

A rheological investigation was conducted on both Mater-Bi^®^ and PHBV at 180 °C to assess their respective flow behaviors. This temperature was specifically chosen to evaluate their suitability for co-extrusion film blowing, particularly considering the inherent thermal mismatch: 180 °C is close to the processing temperature of PHBV but significantly higher than the usual processing temperature for Mater-Bi^®^. Key rheological parameters, including *G′*, *G″*, and η*, were measured to characterize the viscoelastic properties of the polymers under processing-relevant conditions. These parameters provide insights into the polymers’ melt elasticity, viscosity, and overall processability, which are critical for ensuring stable film formation during extrusion. The results, illustrating the frequency-dependent viscoelastic behavior of both materials, are shown in Figure 2.

The rotational rheology data reveal distinct viscoelastic behaviors for Mater-Bi^®^ and PHBV Y1000P, highlighting fundamental differences in their melt responses—factors that critically influence their suitability for film-blowing applications. The Mater-Bi^®^ sample exhibits a highly structured behavior characterized by dynamic moduli several orders of magnitude higher than PHBV. At low frequencies, Mater-Bi^®^ shows a predominant elastic response (*G′* > *G″*), suggesting a stable physical network or high degree of entanglement. However, a crossover occurs, where the loss modulus *G″* becomes dominant, marking a transition to viscous-dominated behavior typical of thermoplastic melts under high-shear conditions. Conversely, PHBV Y1000P behaves as a predominantly viscous fluid across the entire tested range, with *G″* consistently exceeding *G′*. The steep decline of *G′* in the low-frequency terminal region confirms that PHBV chains relax easily, indicating high flowability but significantly lower melt strength compared to Mater-Bi^®^ grade. The viscoelastic profile of Mater-Bi^®^ is particularly favorable for film blowing, where elastic recovery and resistance to deformation are essential for achieving bubble stability and uniform film thickness. As such, the Mater-Bi^®^ grade used for this work had excellent processability, with minimal defects arising from melt instability. In contrast, PHBV Y1000P displays considerably lower elasticity, with *G*′ values significantly below those of Mater-Bi^®^ across the frequency range. As a result, stable film blowing was impossible to achieve with pure PHBV, due to its insufficient elastic recovery and poor resistance to extensional deformation during processing. On the other hand, Mater-Bi^®^ exhibited the opposite trend, confirming its good processability across a wide range of temperatures and strains, which is directly supported by its superior rheological behavior, particularly its high melt elasticity. Mater-Bi^®^ also exhibits high η* across the tested frequency range, which decreases with increasing frequency, a classic shear-thinning behavior typical of polymer melts. This reduction in viscosity at higher shear rates facilitates processing by lowering resistance during extrusion, while the high zero-shear viscosity ensures adequate melt strength. PHBV Y1000P, by contrast, displays substantially lower η* at all frequencies, with a slight shear-thinning behavior. Such low viscosity also indicates low melt strength. As noted earlier, this rheological weakness contributed to the inability to process PHBV as a standalone material via film blowing, due to the onset of bubble instability and premature rupture.

The rheological characterization of Mater-Bi^®^ and PHBV Y1000P confirms the feasibility of combining these two biopolymers through co-extrusion. In the co-extruded multilayer film, Mater-Bi^®^ serves as the support layer, providing mechanical stability and facilitating the film-blowing process due to its superior rheological performance, while PHBV is incorporated as a thin inner layer, contributing its barrier and mechanical properties without compromising processability.

Mater-Bi^®^ acts as a rheological carrier, providing the melt strength (*G′* > *G″* at low frequency) needed to stabilize the bubble and to sustain the biaxial stretching of the thin PHBV layer (*G″* > *G′*). This viscoelastic coupling allows PHBV to orient without rupture, enabling stable co-extrusion despite its low melt strength: the thin PHBV layer behaves as a viscous melt (*G″* > *G′*) with limited molecular orientation, while the thick Mater-Bi^®^ layer functions as an elastic carrier (*G′* > *G″*) with high elastic deformation and molecular orientation.

### 3.2. Differential Scanning Calorimetry

**Figure 3:**
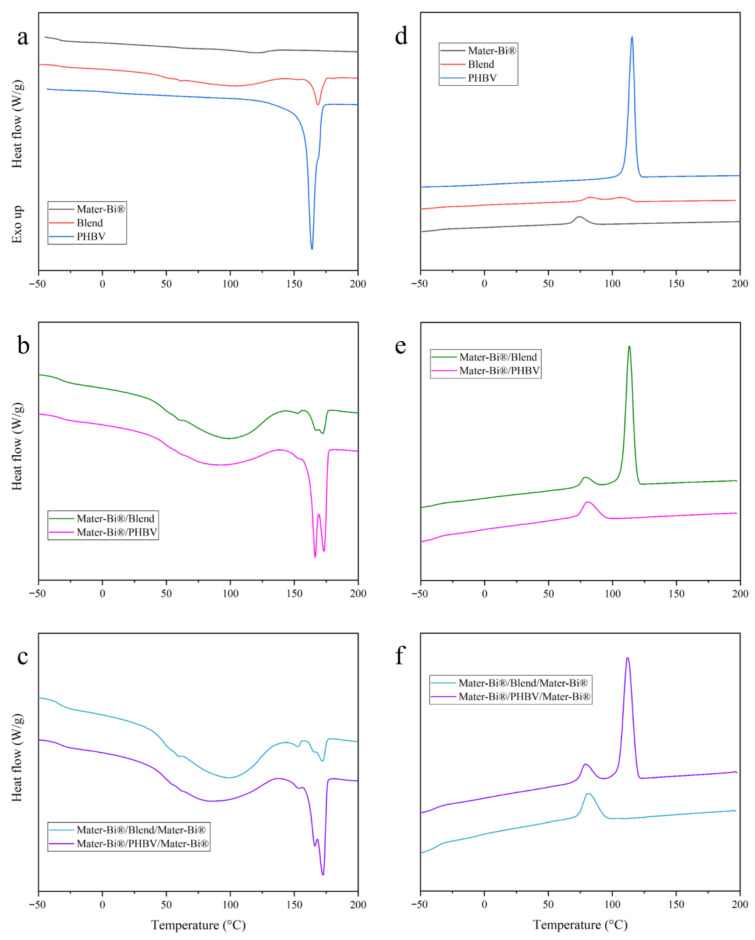
DSC scans of heating for pellet (**a**), bilayer (**b**) and ternary layer (**c**), and cooling for pellet (**d**), bilayer (**e**) and ternary layer (**f**).

The thermal properties of the polymers, in both their raw pellet and final film forms, were investigated using DSC. Figure 3 shows DSC scans for a representative sample and Table 1 summarizes the key thermal transitions extracted for all samples analyzed, including glass transition temperatures (*T*g), process-induced phenomena (*T*pi, Δ*H*pi), crystallization parameters (*T*c, Δ*H*c), and melting behavior (*T*m, Δ*Hm*). In the case of a complex thermal transition, the presence of two reported temperatures indicates two distinct, partially overlapping peaks. If the secondary feature is either a shoulder on the main peak or a visible but less intense peak, the corresponding temperature is reported in parentheses.

For PHBV-containing samples, the degree of crystallinity (*X*c) was also reported, calculated from the melting enthalpy using Equation (3):

(3) $$X_{c}=\frac{\Delta H_{m}}{\Delta H_{m}{}^{\circ}}\cdot 100$$

where ∆*H*m is the measured heat of fusion and ∆*H°*m is the heat of fusion of a 100% crystalline polymer.

#### 3.2.1. Pure Components and Blend Characterization

Mater-Bi^®^ exhibited a glass transition temperature at −34 °C and a broad melting endotherm between 35 and 150 °C (peak at 120 °C, Δ*H*m = 15 J/g). This behavior reflects the complex composition of Mater-Bi^®^, composed of TPS, synthetic polyesters, plasticizers, and additives, resulting in a largely amorphous material with only minor crystalline domains. Crystallization during cooling occurs at 74 °C (Δ*H*c = 10 J/g). Experimental DSC studies reported very low crystallinity for Mater-Bi^®^: Nainggolan et al. measured ~4.25% crystallinity for neat Mater-Bi^®^, decreasing further when reinforced with cellulose fibers [12] while more recent analyses indicate similarly low melting enthalpies (Δ*H*m ≈ 30 J/g), consistent with a largely disordered matrix [32]. For Mater-Bi^®^, given its complex multi-component composition and the absence of a well-defined crystalline reference state, we report enthalpies without converting them to crystallinity percentages.

In contrast, PHBV exhibits semi-crystalline behavior with *T*g of 6 °C. The melting profile showed a double endotherm with the main peak at 164 °C and a shoulder at 168 °C. This double melting is characteristic of the melting–recrystallization mechanism in polyhydroxyalkanoates [33]. In the absence of a reference value for the melting enthalpy at 100% crystallinity (Δ*H°*m) of PHBV, the value of 146 J/g, corresponding to PHB, was adopted [34]. It should therefore be noted that this value represents an upper bound for the true reference enthalpy, since the presence of 3 mol% hydroxyvalerate units in PHBV is expected to disrupt the crystalline order, thereby reducing the melting enthalpy of the fully crystalline material. From the measured melting enthalpy of 94 J/g, we calculated 64% crystallinity. During cooling, PHBV crystallized at 116 °C with an enthalpy of 86 J/g.

Film blowing significantly altered thermal behavior. Mater-Bi^®^ film displayed a large process-induced endotherm at 90 °C (103 J/g) with no distinct melting peak, indicating extensive molecular orientation. PHBV film showed melting at 167 °C with a 172 °C shoulder (89 J/g, 61% crystallinity) and crystallized at 120 °C (4 °C higher than the pellet), suggesting that biaxial stretching facilitates crystallization.

The 80/20 blend film revealed immiscibility through distinct glass transitions: −33 °C for Mater-Bi^®^ and −3 °C for PHBV. The Mater-Bi^®^-rich phase showed a process-induced endotherm at 67 °C (102 J/g), while the PHBV-rich phase displayed a single melting peak at 169 °C (14 J/g), contrasting with the double peak in pure PHBV film. A shoulder is barely visible on the high-temperature side of the peak, suggesting a minor fraction of more highly ordered crystals. Presumably, the shear stress across the interface of PHBV inclusions in the blend is not sufficient to promote the formation of more perfect crystals. During cooling, PHBV-rich phase crystallized at 106 °C (7 J/g) (14 °C below pure film with 93% reduced enthalpy) while Mater-Bi^®^-rich phase crystallized at 82 °C (8 J/g), slightly above the pellet. These shifts indicate interfacial effects despite immiscibility.

#### 3.2.2. Process-Induced Mater-Bi^®^ Crystallinity in Multilayer Blown Film

The DSC analysis also provides critical insights into the effects of the extrusion process on the material structure, depending on composition. All blown film samples containing Mater-Bi^®^ displayed a broad endothermic transition during first heating, spanning roughly 20–140 °C with peak temperatures between 67 and 90 °C. The enthalpy ranged from 58 to 103 J/g depending on composition. The higher value was exhibited by the pure Mater-Bi^®^ drawn film. This transition was absent in the pelletized materials and in the PHBV drawn film. Therefore, it represents process-induced crystallinity generated by the biaxial drawing occurring during the film-blowing process of the Mater-Bi^®^-rich phase. The magnitude of this endotherm (58–103 J/g) was much larger than that observed during subsequent cooling (5–10 J/g), with a recovery of only 9–11% depending on the multilayer structure. This indicates that the oriented morphology generated during film blowing cannot be fully restored through thermal cycling alone. This process-induced crystallinity proved largely irreversible. During cooling in the absence of shear stresses, Mater-Bi^®^ crystallized at 78–82 °C with only 5–10 J/g, representing just approximately 10% recovery of the initial crystallinity. Temperature-modulated DSC studies on oriented biodegradable polymers report that process-induced endotherms are primarily observed in the non-reversing heat flow signal, suggesting a kinetic, rather than equilibrium, nature [35]. Similar endotherms above *T*g in drawn PLA films were attributed to relaxation of oriented chain conformations during heating-induced shrinkage [36]. In our multilayer films, the endotherm peaks (67–90 °C) occur well above Mater-Bi^®^ glass transition (−33 °C), which is consistent with thermally activated endothermic relaxation of the biaxially oriented structure.

#### 3.2.3. PHBV Melting and Crystallization Behavior: Interfacial and Confinement Effects

A critical theoretical question in co-extruded multilayer systems is the adhesion between layers in the absence of tie layers. Since both PHBV and the polyester components of Mater-Bi^®^ are aliphatic polyesters, they possess a degree of thermodynamic compatibility that allows for molecular entanglement at the melt interface during co-extrusion [37,38,39,40,41,42].

This interfacial strength significantly influences the resulting morphology. Pure PHBV film exhibited a melting endotherm with a main peak at 167 °C and a minor shoulder at 172 °C. In contrast, the PHBV contribution in the blend film was characterized by a single melting peak at 169 °C, indicating the loss of the bimodal melting behavior observed in the neat polymer.

Multilayers with pure PHBV layers consistently showed two resolved peaks. The Mater-Bi^®^/PHBV bilayer displayed peaks at 166 °C and 173 °C (34 J/g total), while the trilayer showed 166 °C and 172 °C (23 J/g). The higher-temperature peak is assigned to PHBV crystallites formed at the inclusion interface, where the local shear stress field induces molecular orientation, favoring the development of more ordered and geometrically perfect crystals. The lower-temperature peak, in contrast, is attributed to bulk PHBV located in the interior of the inclusions, where the reduced stress field does not provide sufficient driving force for chain alignment, resulting in a less ordered crystalline morphology. Multilayers with pure PHBV layers showed substantially higher PHBV crystallinity degrees: 58% (Mater-Bi^®^/PHBV bilayer) and 76% (Mater-Bi^®^/PHBV/Mater-Bi^®^ trilayer), compared to 48% in the blended system. We attribute this increase in crystallinity to the interplay between crystallization sequence and melt strength during film blowing. Upon cooling during blowing, PHBV crystallizes at higher temperatures than Mater-Bi^®^, while the latter remains in the melt phase. However, owing to its higher melt strength, Mater-Bi^®^ can sustain significant biaxial stress, thereby imposing an oriented deformation field on the adjacent PHBV layers during their crystallization. This strain-induced crystallization promotes a higher degree of crystallinity in PHBV layers contained in the multilayer films.

Additionally, PHBV crystallization behavior depended strongly on configuration. Pure layers in both bilayer and trilayer structures crystallized at 112–113 °C with substantial enthalpies (36 and 24 J/g). Normalized by PHBV content, these correspond to 90–120 J/g per unit PHBV. The values match or exceed what is observed for pure material. Even the 10 µm confined central layer in the trilayer crystallized efficiently, demonstrating that Mater-Bi^®^/PHBV interfaces provide effective heterogeneous nucleation sites.

On the other hand, when dispersed in the blend, PHBV crystallization was severely hindered (106 °C, 7 J/g in blend film), and no PHBV crystallization was resolved in blend-containing multilayers. During cooling in the absence of external force fields experienced during film extrusion and blowing, molecular-level dispersion restricts chain mobility and eliminates effective nucleation, preventing crystallization at standard cooling rates.

Mater-Bi^®^ showed consistent behavior across samples (78–82 °C, 5–10 J/g), representing partial crystallization due to slow kinetics of this starch–polyester blend. The architectural control demonstrated—with layer interfaces promoting crystallization while dispersion hinders it—provides a route to tailor both thermal and physical properties in biodegradable multilayer packaging films.

### 3.3. Barrier Properties

**Figure 4:**
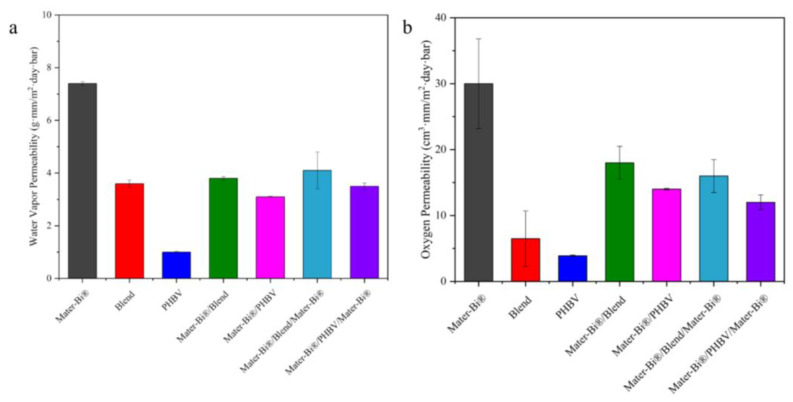
Water vapor permeability (**a**) and oxygen permeability (**b**) of multilayers and neat films.

The effectiveness of a packaging material is critically determined by its ability to serve as a barrier to gas and moisture transmission, which directly impacts the shelf life and quality of packaged products. Therefore, both water vapor permeability (WVP) and oxygen permeability (*OP*) (Figure 4) were assessed.

The WVP of the neat materials demonstrated the superior barrier property of PHBV, which registered a value of 1.0 g·mm/(m^2^·day·bar), whereas the Mater-Bi^®^ films exhibited WVP values of 7.4 g·mm/(m^2^·day·bar) for the 50 µm films. These materials are collectively categorized as hydrophobic by Wang et al. [43], who established a WVP ceiling of 40 g·mm/(m^2^·day·bar) for such polymers.

Remarkably, the introduction of PHBV as a component in multilayer and blend structures successfully reduced the higher WVP observed in the neat Mater-Bi^®^ films. The significantly lower WVP of PHBV places it as an effective moisture barrier component, substantially enhancing the barrier performance of the resultant double-layer and three-layer films. These findings suggest a clear strategy for the design and optimization of multilayer films utilizing PHBV to achieve the high barrier performance necessary for packaging applications that require stringent control over water vapor transmission.

OP highlights the material’s capacity to exclude atmospheric gases, a requirement for preserving sensitive products against oxidative degradation. Similar to the WVP results, both the Mater-Bi^®^/PHBV/Mater-Bi^®^ multilayer and the equivalent blend demonstrated a marked reduction in OP when compared to the neat Mater-Bi^®^ films. This enhanced performance establishes these PHBV-containing films as excellent oxygen barriers, as suggested by the literature [44,45,46]. For the blends, permeability reduction follows a tortuosity-based mechanism consistent with the Nielsen model (Equation (4)):

(4) $$P_{eff}=P_{m}\cdot \frac{1-\phi_{b}}{1+A\cdot \phi_{b}}$$

where *P_m_* is the matrix permeability, A is a factor related to the aspect ratio of the dispersed barrier phase, and $A\cdot \phi_{b}$ is the barrier phase fraction. The experimental data show only partial reductions in both WVP and OP for the blends, indicating that the dispersed PHBV phase increases path tortuosity but does not establish continuous transport resistance. The improvement remains limited, consistent with the sub-linear dependence predicted by tortuosity models.

The multilayer films represent a series resistance, according to (Equation (5)):

(5) $$\frac{1}{P_{eff}}=\sum_{i}\frac{\phi_{i}}{P_{i}}$$

The barrier behavior does not follow a purely series resistance trend. Although multilayers contain a continuous PHBV layer, the 80/20 blend showed OP values comparable or below to those of the bilayer. This indicates that oxygen permeability depends not only on layer thickness and continuity but also on crystallinity and the morphology of the PHBV-rich dispersed phase. Barrier performance therefore results from the interplay of PHBV crystallinity, dispersion state, and layer geometry.

Since transport occurs perpendicular to the layer plane, permeant molecules must sequentially traverse each continuous layer. Even when the PHBV thickness fraction is modest, its low intrinsic permeability dominates the overall transport resistance. The reduction in OP observed for the Mater-Bi^®^/PHBV multilayer films relative to neat Mater-Bi^®^ confirms this series-controlled behavior. The deviation from blend performance cannot be explained solely by volume fraction effects, but rather by the transition from dispersed to continuous barrier morphology. Such enhancement, which is well-established in transport literature, is also mirrored in the mechanical behavior through strain partitioning and interfacial constraint, as described later on.

These findings confirm the potential to optimize the architectural design and composition of the films. By strategically utilizing the superior barrier properties of PHBV (low WVP and OP) within multilayer structures, it is possible to achieve the low vapor and gas transmission rates essential for high-end packaging applications that demand stringent control over environmental factors.

### 3.4. Mechanical Properties

**Figure 5:**
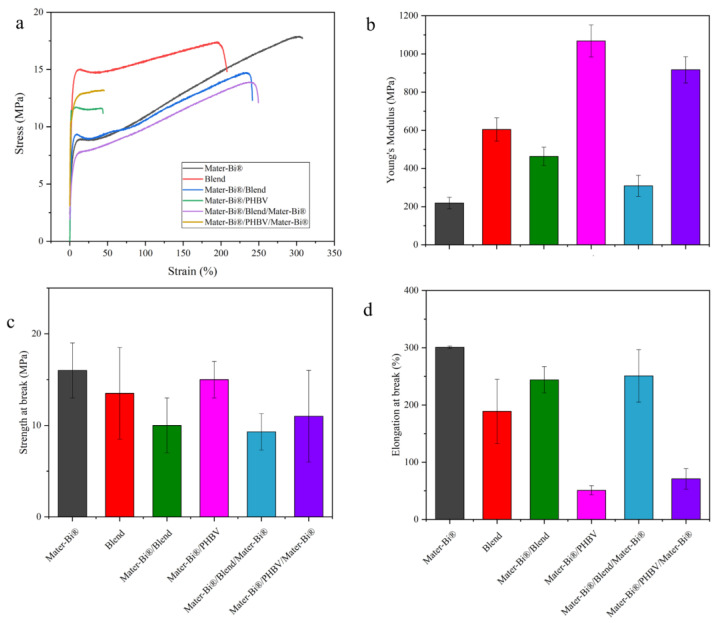
Representative stress–strain curves of neat films and multilayers (**a**); main mechanical properties reported as mean values ± SD: Young’s modulus (**b**), tensile strength at break (**c**), and elongation at break (**d**).

Assessing the mechanical properties of a polymer film is fundamental for determining its suitability for various applications, especially packaging. Films must possess sufficient mechanical strength and elongation to maintain their structural integrity and withstand external forces encountered throughout a product’s lifecycle (e.g., handling, processing, and transportation). To analyze the influence of multilayer film design, tensile mechanical tests were conducted to compare the properties of pure films, blends, and multilayer configurations (Figure 5).

The neat polymers exhibit distinct mechanical profiles: Mater-Bi^®^ is characterized by its high ductility, while PHBV is classified as a rigid and brittle polymer (low ductility and high stiffness). The study of the multilayer structures clearly demonstrates the role of each component in tailoring the final properties: the incorporation of the inherently rigid PHBV polymer significantly increases the film’s stiffness. For instance, the Mater-Bi^®^/PHBV film (either as a blend or as a simple bilayer) exhibits a greater elastic modulus compared to the neat Mater-Bi^®^ film. In addition, the resulting film ductility is highly dependent on the relative proportions of the two polymers: in multilayer configurations, an increase in PHBV content directly leads to reduced ductility and a corresponding increase in brittleness. Conversely, an increase in Mater-Bi^®^ content serves to enhance the overall ductility of the films. These findings confirm that the mechanical profile of the final film can be effectively tuned between the ductile behavior of Mater-Bi^®^ and the rigid behavior of PHBV by controlling the blend ratio or the layer structure. The improved mechanical balance of the multilayers can be interpreted through strain partitioning: ductile Mater-Bi^®^ layers distribute stress and delay micro-cracks in the brittle PHBV layer, while interfacial adhesion constrains localized necking in PHBV and promotes more uniform deformation [47,48,49]. During tensile loading, the more compliant Mater-Bi^®^ layers act as a buffer, effectively distributing stress and delaying the propagation of micro-cracks that typically initiate in the brittle PHBV phase. Furthermore, strong interfacial adhesion (achieved via molecular inter-diffusion) creates a ‘constraint effect’ at the boundary, the Mater-Bi^®^ carrier prevents the localized necking of the thin PHBV layer, forcing it to undergo more uniform deformation. This interaction explains why the multilayer structure can maintain significant stiffness without the premature failure associated with pure PHBV.

### 3.5. Heat-Seal Strength

**Figure 6:**
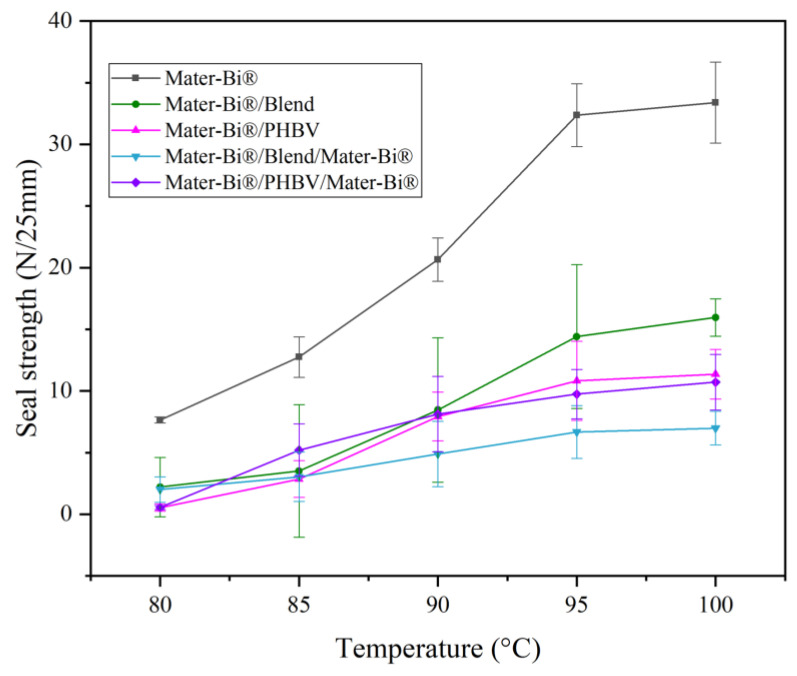
Heat-seal strength values for Mater-Bi^®^ and multilayers.

The seal strength of a packaging film is a critical functional requirement, particularly for high-speed automated processes. A material’s seal strength must be sufficient to maintain package integrity. The presence of a crystalline phase, which interferes with polymer chain mobility during heat application, is often the main factor that inhibits successful sealing. The sealing behavior of the films directly reflects the influence of the blend ratio and multilayer architecture: pure PHBV was found to be non-sealable [50]. This failure is attributed to its high crystalline phase content, which prevents the necessary melt flow and inter-diffusion across the sealed interface. Figure 6 and Table 2 show the experimental results for the evaluation of heat-seal strength. The Mater-Bi^®^/PHBV 80/20 blend (not structured as a multilayer) was also non-sealable, indicating that the presence of the non-sealing PHBV phase within the blend matrix dominated the overall sealing performance. Pure Mater-Bi^®^ films, almost completely amorphous, generally facilitated good sealing. Mater-Bi^®^ was strategically utilized as the outer layer in multilayer structures to enable film sealing ability. The following structures successfully met the 6.5 N/25 mm threshold: Mater-Bi^®^, Mater-Bi^®^/Blend (two-layer structure with a blend core of Mater-Bi^®^/PHBV), Mater-Bi^®^/Blend/Mater-Bi^®^ (three-layer structure with a blend core), Mater-Bi^®^/PHBV (two-layer structure with neat materials), and Mater-Bi^®^/PHBV/Mater-Bi^®^ (three-layer structure). Such results indicate the formation of a robust interfacial assembly. Despite the inherent thermodynamic immiscibility of the two phases, the interfacial stability appears to be governed by a synergistic combination of kinetic, chemical, and morphological drivers, as discussed below. During the co-extrusion process, the elevated temperatures within the feed block and die provide the thermal energy to trigger localized segmental mobility yielding to short-range segmental diffusion across the melt interface. As the melt cools, these inter-diffused chain segments become kinetically trapped, inducing a molecular “anchoring” effect.

From a theoretical standpoint, this sealing mechanism is governed by the reptation of polymer chains across the interface [40,51,52].

While the high crystallinity of pure PHBV restricts the “tube” mobility of its chains, the adjacent amorphous Mater-Bi^®^ phase provides a high-volume fraction of mobile segments that can penetrate the PHBV surface. This asymmetric diffusion allows the Mater-Bi^®^ to act as a molecular “Velcro”, creating entanglements that the rigid PHBV cannot achieve on its own.

The subsequent rapid crystallization of the PHBV phase further locks these diffused segments into the opposing Mater-Bi^®^ matrix. Furthermore, the adhesion of the layers is partially attributed to the chemical constituents of the Mater-Bi^®^ matrix, which typically incorporates PBAT. Both PHBV and the PBAT component of Mater-Bi^®^ are polyesters characterized by ester functional groups (–COO–) along their macromolecular backbones. This structural homology could suggest the formation of intermolecular interactions that prevent delamination. Beyond short-range chain inter-diffusion favored by ester–ester compatibility, a plausible additional contribution may arise from oriented crystalline regions forming near the interface during cooling, acting as anchoring sites. This mechanism is proposed as a hypothesis consistent with the observed macroscopic cohesion. In fact, in addition to the explanation based on chain entanglement and ester linkage homology discussed before, the observed cohesion suggests another bridging mechanism. In particular, as demonstrated by Li et al. [53,54] in their analysis of 3D stitched composites, the introduction of through-thickness reinforcements effectively suppresses the sharp propagation of delamination.

The results confirm that incorporating a layer of Mater-Bi^®^ on the sealing surface is an effective strategy to overcome sealing deficiencies imparted by the PHBV component, thereby enabling the composite to be used in standard heat-sealing applications.

### 3.6. Optical Properties

Optical properties are a fundamental aspect of food packaging, influencing both product presentation and preservation. To assess these qualities, the films were evaluated using colorimetry and by measuring light transmission, which determines the film’s transparency. The transmittance percentage at 560 nm (T_560_) is widely adopted as a standard metric to quantify film transparency, with higher values indicating greater clarity.

**Figure 7:**
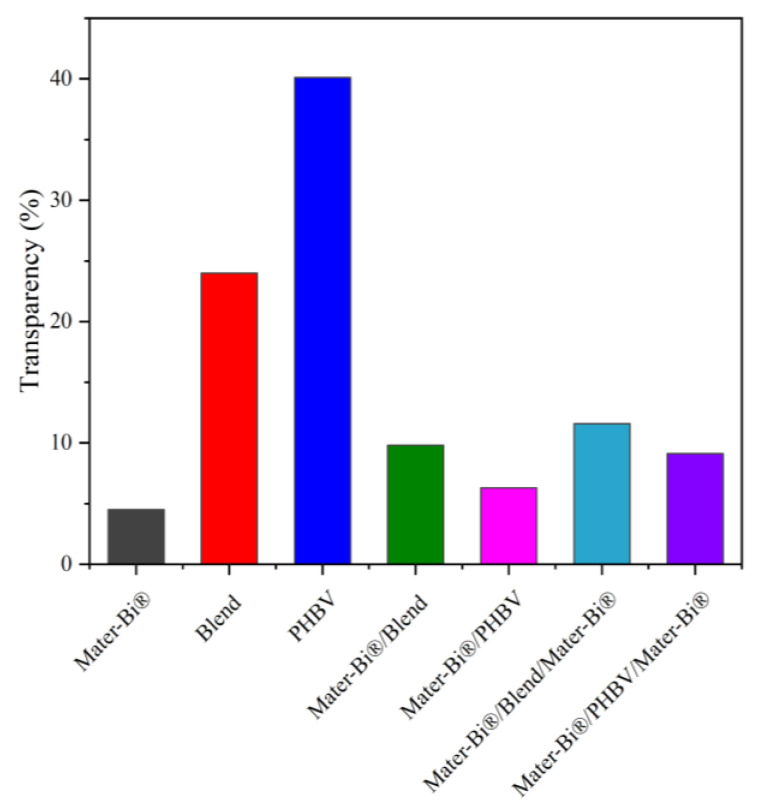
Transparency of neat films and multilayers.

The pure films exhibited distinct optical characteristics: PHBV demonstrated the highest transparency (a *T*_560_ of 40.1% for the tested films, as shown in Figure 7) while pure Mater-Bi^®^ showed low transparency (or high opacity/haze). The optical properties of the blend and multilayer films were primarily driven by the relative content of each polymer and the resulting microstructure: for both the blends and multilayer structures, transparency consistently decreased as the content of Mater-Bi^®^ increased. This is a direct consequence of Mater-Bi^®^ lower inherent transparency. It is worth highlighting that, in general, an increase in crystallinity typically leads to increased haze (light scattering) and a decrease in transparency. This is in contrast to the higher transparency of the PHBV films. However, this high clarity may be due to the small size of the crystalline structures or their homogeneous distribution, which minimizes light scattering. A clear inverse correlation was observed between film thickness and transparency: as the thickness of the films reduced, the transparency increased (as evident in Figure 7). These results indicate that the optical quality of the film can be tailored to meet specific packaging needs, ranging from the high clarity provided by the PHBV-rich films to the light-barrier function offered by the Mater-Bi^®^-rich structures.

### 3.7. Industrial Competitiveness and Benchmarking

To evaluate the industrial viability of the Mater-Bi^®^/PHBV multilayer films, their performance was benchmarked against neat PLA and PBAT—the two most prevalent commercial biodegradable polymers. PLA offers high tensile modulus (1.8–2.6 GPa) but its inherent brittleness (<10% strain at break) and moderate moisture barrier properties (WVP approximately 10 g·mm/(m^2^·day·atm) limit its application in flexible, high-barrier packaging. Conversely, PBAT provides excellent ductility (>500% strain at break) but lacks the gas barrier necessary for food preservation (WVP > 10 g·mm/(m^2^·day·atm). However, a critical distinction for the Mater-Bi^®^/PHBV multilayer system is its OP. As noted in the comparative data reported in Table 3, neat PBAT is a very low barrier to oxygen, making it unsuitable for packaging lipid-containing or oxygen-sensitive foods. On the other hand, although PLA offers a moderate oxygen barrier, it lacks the flexibility required for vacuum or Modified Atmosphere Packaging (MAP) [55,56,57].

In our multilayer design, the continuous PHBV layer acts as a high-density crystalline shield, resulting in a PO_2_ reduction of over 75% compared to neat PBAT and values significantly lower than those of neat PLA. This performance profile allows the Mater-Bi^®^/PHBV film to compete with non-biodegradable commercial laminates (like PE/EVOH/PE) while maintaining a fully compostable end-of-life.

As shown in Table 3, the co-extruded trilayer films developed in this study bridge the performance gap between these materials. By using Mater-Bi^®^ as a ductile “rheological carrier” and PHBV as a continuous crystalline barrier, the resulting composite maintains the flexibility required for industrial conversion while achieving a water vapor barrier that is significantly superior to those of PBAT and PLA.

As reported in the literature (Bugnicourt et al., 2014) [61], the current market price of PHBV remains significantly higher than that of PLA and PBAT. However, our multilayer approach addresses this economic barrier by confining the premium biopolymer to a functional thin layer, achieving a “high-barrier” classification at a fraction of the cost of a monolithic PHBV film. In particular, from a cost perspective, the multilayer architecture offers a strategic advantage over traditional blending. While PHBV is a premium-cost biopolymer, the co-extrusion process allows for its concentration into a singular, ultra-thin functional layer (<15% of total thickness) that provides 100% of the required barrier. This material-efficient approach, combined with the observed natural interfacial adhesion that eliminates the need for expensive tie layers, suggests that Mater-Bi^®^/PHBV multilayer films represent a cost-competitive and technically superior alternative for the next generation of sustainable, high shelf life packaging.

## 4. Conclusions

The findings of this study demonstrate that combining PHBV and Mater-Bi^®^ in multilayer and blend configurations represents a viable strategy for developing sustainable, high-performance packaging materials. A clear correlation was observed between the thermal, mechanical, barrier, and sealing properties of the films, governed primarily by the degree of crystallinity. PHBV’s high crystallinity imparts rigidity and enhances barrier properties but simultaneously reduces chain mobility, resulting in poor sealability and limited ductility. In contrast, Mater-Bi^®^, with its lower crystallinity, exhibits greater flexibility and reliable sealing behavior. Furthermore, the high melt strength of Mater-Bi^®^ allows it to act as a rheological ‘carrier’ and process stabilizer for the bubble during the film-blowing operation. This strategy effectively overcomes the processing instability and narrow thermal window of PHBV—limitations that typically hinder its transformation into flexible films when processed alone or in simple blends.

This complementary relationship enables the design of multilayer structures, such as the Mater-Bi^®^/PHBV/Mater-Bi^®^ configuration, which effectively balances processability, stiffness, ductility, and sealing ability by integrating the distinct advantages of both polymers. Furthermore, an inverse relationship between mechanical and barrier properties was confirmed: increasing PHBV content improved barrier performance (lower WVP and OP) but reduced elongation at break, emphasizing the importance of multilayer architecture in maintaining performance equilibrium. Also in this case, the degree of crystallinity of PHBV is the key: the presence of crystalline impermeable regions significantly reduces the permeability but acts as physical cross-links that severely restrict chain mobility. This results in a significant increase in tensile strength and modulus at the expense of strain at break shifting the material toward a brittle failure mode. The data confirm that an ideal packaging film—one that is simultaneously highly impermeable, flexible, and sealable—cannot be achieved using either PHBV or Mater-Bi^®^ as a mono-material. Furthermore, PHBV alone exhibits low processability, making a stable film-blowing process nearly impossible. Conversely, Mater-Bi^®^ offers the required ductility and sealing ability together with very good processability but lacks the crystalline density necessary for a high-performance barrier. Therefore, an optimal trade-off among barrier efficiency, ductility and sealing ability is best achieved via multilayer structuring, which integrates PHBV’s barrier contribution with Mater-Bi^®^ processability and sealing capability within a fully compostable architecture.

Regarding sealing performance of the films, it was found to be a direct function of the surface layer’s crystallinity and molecular mobility. Heat sealing relies on the inter-diffusion of polymer chains across the interface to form a cohesive bond. In the case of neat PHBV, the high degree of crystallinity acts as a network of physical cross-links that restricts chain mobility even at elevated temperatures. This prevents sufficient chain entanglement, resulting in poor seal strength and brittle failure at the joint. In contrast, Mater-Bi^®^—characterized by lower crystallinity—exhibits the necessary molecular flow to facilitate a robust hermetic seal. To overcome the ideal film dilemma, a Mater-Bi^®^/PHBV/Mater-Bi^®^ multilayer architecture was employed. This configuration utilizes the PHBV or a Mater-Bi^®^/PHBV blend core as a high-efficiency gas barrier while employing Mater-Bi^®^ as the functional “skin” to provide the required ductility and sealing ability.

Optical analysis further revealed that transparency decreases with increasing Mater-Bi^®^ content due to light scattering, allowing compositional tuning for specific applications. Overall, these correlations underscore that the multilayer design effectively exploits the complementary properties of PHBV and Mater-Bi^®^, resulting in films that combine mechanical strength, barrier efficiency, ductility, and sealability. This approach provides a pathway toward the development of multifunctional, biodegradable packaging materials capable of meeting modern industry demands for product protection and environmental sustainability.

This study demonstrates that the Mater-Bi^®^/PHBV/Mater-Bi^®^ configuration enables a performance equilibrium that is difficult to achieve with single-component biopolymers. This synergy makes these films particularly suitable for the following sectors: fresh and perishable food—the core PHBV layer acts as a high-efficiency gas barrier. This is critical for Modified Atmosphere Packaging (MAP) of products such as red meat and fresh pasta. In these applications, maintaining a specific internal atmosphere is essential to inhibit microbial growth and prevent lipid oxidation, thereby extending shelf life without the reliance on synthetic preservatives; dry goods and structural packaging—an advantage of this multilayer design is its tunability. By modulating the thickness and the composition of the PHBV-based inner layer, the structural rigidity of the film can be precisely adjusted. This facilitates the development of stand-up pouches for coffee, grains, or legumes that require both mechanical “stand-up” stability and a high barrier against moisture and oxygen to maintain product crispness and aroma.

While direct food storage trials were beyond the current scope of this study, the measured functional properties provide a strong and quantitative scientific basis for the expected efficacy of these multilayer films in fresh-keeping applications. The substantial reduction in oxygen transmission is particularly relevant for Modified Atmosphere Packaging (MAP) systems, where controlled oxygen levels are critical to suppress aerobic microbial proliferation and mitigate oxidative degradation processes in oxygen-sensitive products, such as fresh pasta and red meat.

Moreover, the multilayer architecture enables a clear functional synergy between components. The intrinsically low water vapor permeability of PHBV limits moisture transfer, thereby reducing dehydration and associated textural deterioration of packaged goods. At the same time, the external Mater-Bi^®^ layers provide excellent heat-seal performance, achieving a seal strength of 10.7 N/25 mm, which surpasses typical industrial benchmarks for hermetic closure in food packaging and ensures package integrity throughout handling and distribution.

While the laboratory results are promising, transitioning to large-scale industrial production involves addressing specific technical hurdles inherent to bio-polyesters.

Narrow processing window: PHBV (as with all polymers of the PHA’s family) is thermally sensitive, possessing a narrow temperature gap between its melting point and the onset of thermal degradation. Successful co-extrusion on industrial lines will require advanced thermal management and precisely calibrated screw profiles to prevent the loss of molecular weight during melt processing.

Interfacial adhesion: although no significant delamination was observed between the PHBV and Mater-Bi^®^ layers within the scope of this study, we acknowledge that the high crystallinity of PHBV and the distinct chemical nature of Mater-Bi^®^ could represent a critical point in different processing conditions or at higher thicknesses. Therefore, monitoring interfacial delamination resistance remains essential for ensuring structural integrity under high mechanical stress.

In conclusion, this study emphasizes that the transition toward sustainable packaging is not merely a matter of replacing conventional plastics with single-component biopolymers. Instead, it requires a strategic architectural approach. By utilizing a multilayer design, we effectively overcome the inherent weaknesses of PHBV (such as brittleness and poor sealing ability) while fully exploiting its superior barrier properties. This research provides a clear pathway for the development of multifunctional, biodegradable materials capable of meeting the rigorous protection standards required by the modern food industry while adhering to environmental sustainability goals.

## Figures and Tables

**Table 1:** Thermal properties of the pellets.

Sample		*T*g (°C)	*T*pi * (°C)	Δ*H*pi * (J/g)	*T*c (°C)	Δ*H*c (J/g)	*T*m ** (°C)	Δ*H*m (J/g)	*X*c (%)
Mater-Bi^®^ pellets		−34	-	-	74	10	120	15	-
PHBV pellets		6	-	-	116	86	164 (168)	94	64
Mater-Bi^®^ 50 μm		−34	90	103	80	10	-	-	-
PHBV 50 μm		9	-	-	120	93	167 (172)	89	61
Blend 50 μm	(Mater-Bi^®^)	−33	67	102	82	8	-	-	-
	(PHBV)	−3	-	-	106	7	169	14	48
Mater-Bi^®^/Blend 50 μm	(Mater-Bi^®^)	−33	79	97	80	10	-	-	-
	(PHBV)	−3	-	-	-	-	(166) 172	7	60
Mater-Bi^®^/PHBV 50 μm	(Mater-Bi^®^)	−32	86	58	78	5	-	-	-
	(PHBV)	−2	-	-	113	36	166 173	34	58
Mater-Bi^®^/Blend/Mater-Bi^®^ 50 μm	(Mater-Bi^®^)	−34	96	88	80	10	-	-	-
	(PHBV)	−2	-	-	-	-	(165) 172	5	70
Mater-Bi^®^/PHBV/Mater-Bi^®^ 50 μm	(Mater-Bi^®^)	−32	82	63	79	6	-	-	-
	(PHBV)	-	-	-	112	24	(166) 172	23	76

* process induced values; ** the *T*m values in brackets refer to not completely solved or secondary peaks.

**Table 2:** Heat-seal strength determination test of neat films and multilayers.

Sample	Load (N/25 mm)	Optimal Temperature	Remark
Mater-Bi^®^	33 ± 3.3	85–100	Sealable
Blend	0.75 ± 0.3	-	Not sealable
Mater-Bi^®^/PHBV/Mater-Bi^®^	10.7 ± 2.3	90–100	Sealable
Mater-Bi^®^/PHBV	11.0 ± 2.0	90–100	Sealable
Mater-Bi^®^/Blend/Mater-Bi^®^	6.9 ± 1.4	95–100	Slightly sealable
Mater-Bi^®^/Blend	16.0 ± 1.5	90–100	Sealable

**Table 3:** Properties of neat PLA, neat PBAT, and Mater-Bi^®^/PHBV Trilayer, in comparison.

Property	Neat PLA *	Neat PBAT *	Mater-Bi^®^/PHBV Trilayer
WVP [g·mm/(m^2^·day·bar)]	10 [58]	13 [59]	3.5
OP [cm^3^·mm/(m^2^·day·bar)]	35 [60]	58 [59]	12
Strain at break (%)	6–16 [55]	388–1250 [55]	69
Tensile modulus (MPa)	1800–2600 [55]	30–130 [55]	917

* Values available in cited literature.

## Data Availability

The original contributions presented in this study are available upon inquiries directed to the corresponding author.
